# Dangers from Impure Water

**Published:** 1885-01

**Authors:** 


### DANGERS FROM IMPURE WATER.

Too much reliance is placed on the senses of taste, sight and smell in determining the character of drinking water. It is a fact that has been repeatedly illustrated that water may be odorless, tasteless and colorless, and yet be full of danger to those who use it. The recent outbreak of typhoid fever in Newburg, N. Y., is an example, having been caused by water which was clear and without taste or smell. It is also a fact that even a chemical analysis sometimes will fail to show a dangerous contamination of the water, and will always fail to detect the specific poison if the water is infected with discharges of an infectious nature. It is therefore urged that the source of the water supply should be kept free from all possible means of contamination by sewage. It is only in the knowledge of perfect cleanliness that perfect safety is guaranteed.

The local European volunteer health commission in Alexandria, where the cholera has been raging along back, is unearthing, according to the *Sanitary News*, some very unsanitary conditions in that city. They have found a large native cemetery, underneath which runs a canal, with which communicates a well, the water of which is used to wash dead bodies. A drinking fountain adjoins this well, and the canal is the water supply of a crowded portion of the town. In the mosques are stagnant pools of water used for ablutions prescribed by religious belief, the water in which, being unchanged, gets indescribably foul. Such nuisances are difficult to abate because of religious prejudices. Is it any wonder that pestilential disease attacks such a locality?

